# Flavonoids as Modulators of Potassium Channels

**DOI:** 10.3390/ijms24021311

**Published:** 2023-01-09

**Authors:** Monika Richter-Laskowska, Paulina Trybek, Domenico Vittorio Delfino, Agata Wawrzkiewicz-Jałowiecka

**Affiliations:** 1 The Centre for Biomedical Engineering, Łukasiewicz Research Network—Krakow Institute of Technology, 30-418 Krakow, Poland; 2 Faculty of Science and Technology, University of Silesia in Katowice, 41-500 Chorzów, Poland; 3 Department of Internal Medicine, Università degli Studi di Perugia, 06123 Perugia, Italy; 4 Department of Physical Chemistry and Technology of Polymers, Silesian University of Technology, 44-100 Gliwice, Poland

**Keywords:** potassium channels, flavonoids, potassium channel modulators, natural substances, Kv channels, Kir channels, KCa channels, K2P channels

## Abstract

Potassium channels are widely distributed integral proteins responsible for the effective and selective transport of $K^{+}$ ions through the biological membranes. According to the existing structural and mechanistic differences, they are divided into several groups. All of them are considered important molecular drug targets due to their physiological roles, including the regulation of membrane potential or cell signaling. One of the recent trends in molecular pharmacology is the evaluation of the therapeutic potential of natural compounds and their derivatives, which can exhibit high specificity and effectiveness. Among the pharmaceuticals of plant origin, which are potassium channel modulators, flavonoids appear as a powerful group of biologically active substances. It is caused by their well-documented anti-oxidative, anti-inflammatory, anti-mutagenic, anti-carcinogenic, and antidiabetic effects on human health. Here, we focus on presenting the current state of knowledge about the possibilities of modulation of particular types of potassium channels by different flavonoids. Additionally, the biological meaning of the flavonoid-mediated changes in the activity of $K^{+}$ channels will be outlined. Finally, novel promising directions for further research in this area will be proposed.

## 1. Introduction

The potassium channels are transmembrane tetrameric proteins allowing for a fast (over $10^{6}$ ions per second) and selective transport of $K^{+}$ ions across the biological membranes down their electrochemical gradient [1]. Although the $K^{+}$ channels are related members of one protein family, they can be divided into several groups according to the structural and mechanistic differences [2,3,4,5]. First, one can discern the voltage-regulated channels (Kv) and the $Ca^{2+}$-regulated $K^{+}$ channels (KCa), being structurally distinguished by six transmembrane domains (TMDs). Another group is formed by the “leak”, which is the double pore $K^{+}$ channels (K2P) composed of four TMDs. The last group is constituted by the inward rectifier potassium channels (Kir), having two TMDs.

The activity of the $K^{+}$ channels plays a pivotal role in a myriad of cellular processes, for instance, volume regulation, proliferation, hormone secretion, neurotransmitter release, and modulation of potential in electrically excitable and non-excitable cells [1]. Due to their fundamental physiological roles, they appear promising drug targets for many diseases ranging from cardiovascular, metabolic, and neurodegenerative disorders to cancers [6,7,8,9,10,11]. The potential therapeutic benefits of the potassium channels targeting by pharmacological agents depend on the efficacy, strength, and selectivity of the interaction between an active compound and a particular ion channel subtype. It is also challenging to introduce such a channel modulator, which would be delivered or act only within a pathological tissue and would not exhibit any harmful side effects.

The mentioned issues give rise to a growing trend in the rational design of novel $K^{+}$ channels’ regulators which requires multidisciplinary investigations among different scientific areas, such as medicinal chemistry, molecular biochemistry, bioinformatics, big data processing, bioengineering, genomics, proteomics, and metabolomics [7,11]. A promising approach is to improve the pharmacokinetics of existing channel-modulating substances such as antibodies, venom peptides, nutraceuticals or medicinal plants [11,12] belonging to the traditional medicine defined by the WHO as “...the sum total of knowledge, skills and practices based on the theories, beliefs and experiences indigenous to different cultures that are used to maintain health as well as to prevent, diagnose, improve or treat physical and mental illnesses...” [13].

Throughout human history, plants and natural products have been the source of effective agents to cure illnesses and improve health. These plant-based traditional curative systems are still widely used and continue to play an essential role in primary health care. Traditional medicines over the years have proved to be an invaluable guide in the current screening of bioactive molecules for therapeutic applications [14] and still are widely used as modern pharmaceuticals or health-beneficial nutrients [15,16,17,18,19]. From a chemical point of view, natural products can be classified based on their biosynthetic origin as (1) polyketides, (2) shikimic-acid-derived natural products, (3) terpenes, (4) glycosides and (5) alkaloids. Among them, we have focused our attention on flavonoids belonging to the group of shikimic-acid-derived natural products [20].

Flavonoids comprise a wide group of polyphenolic compounds of plant origin, which exhibit anti-oxidative, anti-inflammatory, anti-mutagenic, and anti-carcinogenic properties, as well as the capability to regulate the functioning of key cellular enzymes [21,22]. Flavonoids can be divided into subclasses such as flavones, flavonols, flavanols, flavanones, isoflavones, anthocyanidins, and chalcones. Due to their ability to modulate cell physiology, they have a clinically proven positive impact in counteracting a plethora of health problems such as cardiovascular and metabolic diseases, or other inflammation-related pathologies, including cancers, as summarized in [17].

In this work, we will enlighten the molecular aspects of the impact of flavonoids on living cells via the $K^{+}$ channels’ modulation. The interactions of flavonoid molecules with specific subtypes of potassium channels (direct or indirect), and the consequent alterations of their transport properties, can be a crucial factor underlying the observed changes in flavonoid-stimulated cells’ biology. It inspired us to summarize in this review the current state of knowledge about the impact of flavonoids on the activity of potassium channels. This work is arranged according to the main subgroups of these channels. It can provide a valuable contribution from the perspective of indication of the most promising channel-targeting flavonoids and analysis of their derivatives in future research. This overview also outlines the promising directions for further in-depth analysis, considering the physiological effects of flavonoid-induced changes in $K^{+}$ channels’ activity.

## 2. Kv Channels

Voltage-gated potassium (Kv) channels are the largest ion channel family in the human organism consisting of 40 members divided into 12 subfamilies Kv1–Kv12. They have many physiological functions such as shaping action potentials, maintenance of membrane potential, neuronal repolarization, modulation of $Ca^{2+}$ signaling, cell volume regulation and control of cellular proliferation and migration [23,24]. Depending on their functionality and location, they are able to pass the current in and out of a cell in response to a change in transmembrane electric potential.

In the following subsections, we discuss the influence of flavonoids on the activity of particular subtypes of the Kv channels. The key information is presented in Table 1 and Table 2. Table 2 refers only to the modulation of the Kv 11.1 channels. Thus, it is presented in the Section 2.5, which describes the effects of flavonoids on these channels.

### 2.1. Kv1.3 Channel

Kv1.3 channels are activated, as all the other voltage-gated potassium channels, by a change of the membrane potential (membrane depolarization). Although they are expressed in many different tissues of the human body, they play the most prominent role in the T lymphocytes [50,51] where they are responsible for cell activation. Accordingly, the inhibition of these ion channels results in the inactivation of the T cells, which promotes immune suppression. It makes the Kv1.3 channel a therapeutic target for the treatment of such diseases as sclerosis, type 1 diabetes, and rheumatoid arthritis [6,50,52]. The recent findings suggest that an increased expression of these channels is required to induce apoptosis in the cancer cells. Therefore, the Kv1.3 channels are a new molecular target in both the diagnostics and therapy of some oncological diseases (e.g., breast cancer, colon cancer) [53].

Throughout the years, the group of Teisseyre investigated the impact of a large number of different flavonoids on activation of the Kv1.3 channels, both in normal and cancer T cells [53]. They discovered the inhibitory effects of genistein, a popular tyrosine kinase inhibitor, which suppresses the activation of the Kv1.3 channel in a concentration-dependent manner with the half-blocking range of $IC_{50}$ = 30–60 μM [25]. These results are consistent with the previous study presented in [54]. Moreover, Teisseyre et al. observed that under the influence of daidzein, which is a structural analog of genistein, the ion channel’s activity remains unaffected. They further demonstrated that resveratrol (a non-flavonoid polyphenol) is able to decrease the activity of the Kv1.3. channel. This effect is slowly reversible, and it is exerted in a concentration-dependent manner. At the same time, it turned out that the co-application of this polyphenol with genistein did not significantly change the suppression effect of resveratrol. Since in the previous study [25], it was demonstrated that genistein has a similar impact on the Kv1.3 channel as resveratrol, the authors suggested that the inhibitory effects of these compounds are independent of each other, and they interact with different binding sites of the channel.

In the following experiments [30], the influence of four flavonoids: aromadendrin, naringenin and its two derivatives, naringenin-4${}^{\prime }$,7-dimethylether and naringenin-7-methyl-ether, on the Kv1.3 channel activity was investigated in human T lymphocytes isolated from peripheral blood. The single-channel patch-clamp traces revealed that naringenin and aromadendrin did not reduce the Kv1.3 current at the concentration of c=30 μM. On the contrary, the two investigated derivatives of naringenin enabled us to reduce the ionic currents at the same concentrations, and the most effective inhibitory effect was observed for the naringenin-4${}^{\prime }$,7-dimethylether. The authors suggested that the suppressing capabilities of the naringenin-related methylated compounds are due to the presence of one or two methoxyl groups in their structure, which possibly interact in some, yet unknown, way with the Kv1.3 channel protein. Nevertheless, although the methylated versions of naringenin—naringenin-4${}^{\prime }$,7-dimethylether and naringenin-7-methylether—are quite effective blockers of the Kv1.3 channel, they do not enable the complete channel inhibition [30].

In contrast, in [32], it was discovered that 8-prenylnaringenin blocks completely the Kv1.3 channel at c=10 μM. The presence of the prenyl group is anticipated to promote the inhibitory abilities of this compound. Indeed, a few years later, it was demonstrated that other compounds of such structural characteristics, xanthohumol and isoxanthohumol, are able to effectively suppress the activity of the Kv1.3 channel [31]. Although xanthohumol turned out to be slightly more effective in decreasing the ion current, the administration of neither of the investigated compounds led to a complete channel inhibition at the concentration c=30 μM. Even though xanthohumol and isoxanthohumol are less potent in inhibition of the Kv1.3 channel than 8-prenylnaringenin, they are much more effective than the other natural plant-derived compounds such as genistein and resveratrol. Therefore, these results confirm the hypothesis that prenylated flavonoids are much more effective in blocking the Kv1.3 channel due to the presence of the prenyl group, which facilitates the non-conducting state of the channel. To further investigate this problem, Teisseyere and his collaborators decided to make a comparative study between natural, plant-derived flavonoids and those which possess also a prenyl group in their structure [26]. They found that pure flavonoids such as baicalein, wogonin, and luteolin were ineffective. In contrast, two other non-prenylated compounds, acacetin and chrysin, were able to suppress the single-channel current regardless of the absence of the prenyl group in its structure. However, the most potent inhibition was obtained during the application of the 6-prenylnaringenin (6–PR), which suppresses the ion channel in a concentration-dependent manner with $IC_{50}=5.8\;\mu M$. Once again, it suggested that the mechanism responsible for the effective inactivation of the channel is based on interactions of the prenyl group with the channel protein. The authors compared the obtained results regarding the modulation of the Kv1.3 by acacetin with another similar work published by Zhao et al. [27]. They found that there exists a significant discrepancy between their findings and those obtained in [27]. According to Teisseyere and his collaborators, the half-blocking concentration for this compound is approximately equal to $IC_{50}$ = 30μM. In contrast, in [27], it was reported that $IC_{50}=21\;\mu M$. The authors of [26] attributed this difference to the time of the ion channel exposition to this flavonoid. In the experiment performed by the Teisseyere group, the incubation in acacetin dwelled for no longer than 5 min, and its effect on the channel was fully reversible. On the contrary, Zhao and his collaborators exposed the Kv1.3 channel to this compound for at least 15 min, and its effects turned out to be only partially reversible. It suggests that the inhibitory effect of acacetin can change with time, and it may exhibit cytotoxicity at higher concentrations. The wide selection of the examined compounds in the work of Teisseyre [26] allowed the authors to draw the conclusion that there is no correlation between the inhibitory abilities of the flavonoids and their cytotoxicity (at least at the investigated type of cells).

In the most recent study [28], the Teisseyere group investigated the impact of the different flavonoids co-applied with statins, which alone had turned out to be effective in blocking the Kv1.3 channel [55]. Their findings showed that in most cases, modulation of the ion channel with a flavonoid accompanied by a statin is more potent than the administration of this flavonoid alone. The results also demonstrated that the inhibitory effects are not always additive. Therefore, the mechanism of the ion channel’s modulation is complex, and the observed channel’s transport capability strongly depends on the chosen proportions in a mixture of statins and flavonoids. The blocking effects of simvastatin and mevastatin co-applied with 8-prenylnaringenin and simvastatin with 6-prenylnaringenin were significantly more potent than predicted by the simple additive model. On the contrary, the inhibition of simvastatin with xanthohumol and acacetin turned out to be notably weaker than expected by a simple addition.

Another group of flavonoids, chalcones, being derivatives of khellinone comprising two aryl rings linked by an α,β-unsaturated ketone, also exert the inhibitory effect on the Kv1.3 channel. Although the khellinone itself cannot be considered a very potent inhibitor of this ion channel with the half-blocking concentration of $IC_{50}=45\;\mu M$, the $IC_{50}$ is substantially lower for its dimers [56,57]. Thus, for clarity, khellinone and its dimer are not elucidated in Table 1. Nevertheless, the complete summary of the Kv1.3 inhibiting properties of this compound and its derivatives is clearly presented in Table 1 in [57]. Considering other chalcones, recent studies show that also Licochalcone A is effective in blocking the Kv1.3 channel [33]. Only the concentration of c≈0.8μM was enough to reduce the ion channel activity by half.

### 2.2. Kv1.5 Channel

The channels belonging to the Kv1.5 family are expressed in many tissues of the human body [58,59]. The greatest attention of the scientists is, however, focused on its expression in the heart. It has been demonstrated [60,61] that the Kv1.5 channel conducts the ultra-rapid delayed rectifier current $I_{Kur}$, which plays an important role in shaping the atrial action potential (AP) repolarization [62]. The studies suggest that the inhibition of this channel can contribute to the prolongation of the AP duration and, by this, stop the atrial fibrillation (AF) [63]. It is important to note that although the Kv1.5 channel is present in the atria, it is not expressed in the ventricular muscle in the heart. These two factors, i.e., provision of the current driving the AP and selective expression of this ion channel (its presence in the atria and absence in the ventricle), consider the Kv1.5 channels as a potential target for the treatment of the cardiac arrhythmia [64,65,66]. For this reason, it would be useful to find new efficient inhibitors of the Kv1.5 channel.

In [35], Wang et al. studied the inhibitory effects of the hesperetin on the $I_{Kur}$ through the Kv1.5 channels expressed in the HEK 293 cells. It turned out that although externally applied hesperetin can significantly suppress the ultra-rapid delayed rectifier $K^{+}$ current in the concentration-dependent manner with $IC_{50}=23.15\;\mu$M, the presence of this flavonoid in pipette solution yielded no effect. The authors concluded that hesperetin interacts with a channel protein only from the exterior. They also observed inhibition of the $I_{Kur}$ along with the suppression of the Kv1.5 channel. However, the study does not provide any information about the action of this flavonoid on the atrial action potential. Thus, it remains unclear whether hesperetin can induce the termination of atrial fibrillation. Another study [38] investigated the impact of hesperetin on the expression of the Kv1.5 channels in coronary arteries of diabetic and non-diabetic rats. The known fact is that diabetes downregulates the expression of these channels in arterial myocytes. Therefore, it would be beneficial to find a biochemical agent which enhances its expression to the proper level. According to [38], although hesperetin has no impact on the expression of Kv1.5 ion channels in arterial myocytes, it increases the expression of Kv1.2 channels which is also desirable during the treatment of diabetic patients.

In [36], Yang et al. investigated the effect of quercetin on $I_{Kur}$ conducted by the wild-type (WT) and mutant (1502A) human Kv1.5 channels. The measured traces obtained from the patch–clamp experiment revealed no effect of quercetin on the mutated Kv1.5 channels’ functioning. However, significant enhancement of ionic currents in the presence of quercetin was observed for the WT channels (with $EC_{50}=37$ μM). These findings allowed us to conclude that quercetin binds preferentially to neutral amino acid I502, which is located in the S6 helix of the Kv1.5 channel. The observed increase in activation of the Kv1.5 cannot be beneficial in the AF treatment during the early phase of this disease. However, it turns out that the chronic AF results in the reduced expression of Kv1.5 α subunits and prolongation of the action potential [67]. It is, therefore, crucial to increase the $I_{Kur}$ at this stage of the disease. From this perspective, quercetin may be used in the treatment of late phase and chronic AF due to its ability to activate the Kv1.5 channels. Another study [37] showed that quercetin can reverse the inhibition of the Kv1.5 current, primarily induced by the monocrotaline, which generates pulmonary arterial hypertension (PAH) in rats. It indicates another potential application of this flavonoid in treatment of patients suffering from cardiovascular diseases. In turn, very weak inhibitory effects of quercetin were demonstrated in [38] based on the analysis of the Kv1.5 channel stimulation by quercetin and its methylated derivatives (3,7,3${}^{\prime }$,4${}^{\prime }$-tetramethylquecertin, 3,5,7,3${}^{\prime }$,4${}^{\prime }$-pentamethylquecertin) in HEK 293 cells. It turns out that suppression of the Kv1.5 channel activity by 3 and 10 μM quercetin is only weak by ≈3.0±1.8% and ≈5.2±3.1%, respectively. Effects of 3,5,7,3${}^{\prime }$,4${}^{\prime }$-pentamethylquecertin are similar to those induced by pure quercetin; i.e., at 3 and 10 μM, it decreased the current by 3.4 ± 2.4% and 8.3 ± 2.5%. The situation notably changes in the case of the application of 3,7,3${}^{\prime }$,4${}^{\prime }$-tetramethylquecertin at 3 and 10 μM: it inhibited the current by 12.1 ± 2.2% and 20.5 ± 5.2%.

In [39], Choi and his colleagues decided to analyze the impact of (−)-epigallocatechin-3-gallate (EGCG) (the main polyphenolic component of green tea) on the ion currents through Kv1.5 channels expressed in Chinese hamster ovary cells (CHO). They observed the significant downregulation of this ion channel in the presence of this flavonoid, which occurs in a concentration-dependent manner. As it turned out, this inhibition was not suppressed by the protein tyrosine kinase, tyrosine phosphatase and protein kinase C inhibitors. The more profound kinetic analysis of the currents revealed that EGCG interacts directly with multiple states (conformations) of the Kv1.5 channel. It preferentially binds to the channel in the closed state, and blocks it by pore occlusion during the depolarization.

Noguchi et al. [40] examined the inhibitory effect of isoliquiritigenin (ISL) flavonoid contained in licorice on the Kv1.5 channel expressed in the CHO cell line. They observed the mediatory suppression effect of this compound on this ion channel. The application of 100 μM ISL at a membrane potential of 40 mV inhibited the Kv1.5 $I_{Kur}$ current by 38.3%.

In [68], the authors investigated the effect of apigenin on pulmonary hypertension. Although this natural compound does not affect the value of a Kv1.5 ion current, it increases its expression in the pulmonary artery smooth muscle cells (PASMC) of hypoxia-exposed rats, stimulating their apoptosis. These results provide promising therapeutic targets for the treatment of pulmonary hypertension. Another study analyzing results obtained from the patch-clamp experiment performed on HEK 293 cells revealed the weak inhibitory effect of the apigenin on the Kv1.5 channel in the presence of c=3 μM (4.6±2.5%) and c=10 μM (11.1±2.9%) of this flavonoid. Larger effects were observed for the double methylated compound, 7,4${}^{\prime }$-dimethylapigenin, which can suppress the ion current by 16.4 ± 3.1% and 28.8 ± 6.0% at the same concentrations. However, the major reduction of the channel’s activity was observed after application of 5,7,4${}^{\prime }$-trimethylapigenin, which occured in a concentration-dependent manner with $IC_{50}=6.2\;\mu M$. In that case, the current was inhibited by 28.9 ± 2.4% with c=3 μM and 70.2 ± 2.8% with c=10 μM. The more profound analysis of the blocking properties of this flavonoid revealed that it binds mainly to open channels. The inhibition efficacy of 5,7,4${}^{\prime }$-trimethylapigenin on hKv1.5 was also confirmed in the human atrial myocytes $I_{Kur}$, which suggests that the Kv1.5 α subunit is the dominant target for the drug (channel blocker) needed in the treatment of atrial fibrillation.

In [42], Li et al. noted the inhibitory impact of the acacetin on the ultra-rapid delayed rectifier current $I_{Kur}$ and Kv1.5 current during the patch-clamp experiment performed on the atrial myocytes. They concluded that acacetin decreases the $I_{Kur}$ and downregulates other important cardiac currents (such as transient outward $I_{to}$ and acetylcholine-activated $I_{KACh}$$K^{+}$ currents), which altogether have a significant impact on the prolongation of the action potential. Several years later, they carried out the analogous experiment on the human HEK 293 cell line [41], which is best suited for investigations on the molecular mechanisms of channel binding with flavonoids. They once again confirmed the inhibitory properties of acacetin, which blocks the $I_{Kur}$ in a use- and frequency-dependent manner. They found that this flavonoid binds to channels in their open or closed conformations. The acacetin-mediated blocking of the open hKv1.5 channels is mediated by binding this flavonoid to the S6 channel domain.

In [34], it is reported that myricetin can exert beneficial anti-arrhythmic effects via Kv1.5 channel regulation. The experiments on the HEK 293 cells showed that this drug enables effective blocking of the channel and inhibits the $I_{Kur}$.

### 2.3. Kv2.1 Channel

The voltage-dependent potassium channels Kv2.1 are expressed both in the central and peripheral nervous system of mammals where they are predominant mediators of the delayed rectifier current [69,70,71]. They play a prominent role in shaping neuronal excitability [72] and in the glucose-stimulated insulin secretion [73], which makes this channel a promising target in treatment of diabetes.

In [46], the authors showed an inhibitory impact of genistein on the activation of the Kv2.1 channels. The analysis of the data obtained from the patch-clamp technique on HEK293 cells revealed that in the presence of this compound, the ion channel is inhibited in a concentration-dependent manner. The more profound kinetic analysis showed that genistein shifted the voltage dependence of channels’ activation and inactivation to membrane hyperpolarization. It also accelerated the closed-state inactivation and delayed the recovery from inactivation.

In [47], Gu with his colleagues demonstrated the inhibitory impact of (−)-naringenin 4,7-dimethyl ether ((−)-NRG-DM) on the Kv2.1 channel expressed in the CHO cells. They reported that it suppresses the ion current in a concentration-dependent manner with $IC_{50}\approx$ 21 μM and shifts half-maximal voltage toward the higher potentials.

The authors of the article [37] found that, similarly as in the case of Kv1.5 channels, quercetin can prevent the inhibition of the Kv2.1 currents in the pulmonary artery smooth muscle cells (PASMC) of rats pretreated with the monocrotaline.

In [40], the authors studied the impact of licorice and isoliquiritigenin on $I_{Kur}$ mediated by Kv2.1 expressed in H9c2 cells derived from rat cardiac myoblasts. They discovered the blocking impact of this compound on the $I_{Kur}$ with $IC_{50}=0.11\;\mu M$, making it one of the strongest inhibitors of this current.

### 2.4. Kv4 Channels

The Kv4 (Shal) channels are widely expressed in the neurons of different animals [74,75,76]. They mediate the fast A-type $K^{+}$ currents and are thought to be responsible for the fundamental electrical properties of nerve cells.

There is not much information about the modulation of these types of ion channels by the flavonoids. In [77,78], it was shown that pinocembrin upregulates the expression of Kv4.2 channels, which can be beneficial in the treatment of ventricular arrhythmia. A little bit more is known about Kv4.3, which according to [48] can be blocked by genistein and, to a smaller extent, by daidzein. The analysis of the Kv4.3 recordings obtained by the patch-clamp technique in the CHO cells revealed that genistein inhibits the current in a reversible and concentration-dependent manner with $IC_{50}\approx 125\;\mu M$. Moreover, it was found that this inhibition is direct: genistein downregulates the activity of the Kv4.3 channel by binding to the closed-inactivated state of the channel, and these interactions are definitely not mediated by the protein tyrosine kinase mechanism. This research group also investigated the impact of the other flavonoids on the Kv4.3 channel: daidzein and genistin, which are the structural analogs of genistein. Their results showed that although daidzein was able to downregulate the channel in a concentration-dependent manner, the complete inhibition could not be achieved. On the other hand, the presence of genistin had no effect on the activation of this ion channel. Another study [38] showed that 5,7,4${}^{\prime }$-trimethylapigenin is an effective blocker of the Kv4.3 channels in the human atrial myocytes. Thus, it may contribute to the prolongation of the atrial action potential duration needed for the treatment of the atrial fibrillation. The other studies revealed that also (−)-epigallocatechin-3-gallate [44] and naringenin [45] exert a mild inhibitory impact on the Kv4.3 channels.

### 2.5. hERG Channels

The *ether-à-go-go-related* hERG ion channel (Kv11.1 channel), similarly to the already mentioned Kv1.5 channel, is responsible for the electrical activity of the heart. It regulates the cardiac action potential by mediating the repolarizing current [79,80].

The impact of different flavonoids on the hERG channels (Kv11.1 channels) has been already discussed in the review [81]. Here, we will briefly summarize the information gathered by the authors of that paper and supplement it with the recent advancements in this field (Table 2).

One of the most important studies concerning the modulation of the hERG channel was performed by Zitron et al. [82] in 2005, who screened a large number of flavonoids for their inhibitory abilities. Based on the patch-clamp recordings, they found that the most potent inhibitors of the Kv11.1 channels are naringenin, morin, and hesperetin. Naringenin blocked the hERG channel expressed both in the Xenopus laevis oocytes and HEK 293 cells with half-blocking concentrations $IC_{50}=102\;\mu M$ and $IC_{50}=36.5\;\mu M$, respectively. A more detailed analysis revealed that the channels are blocked in the open and inactivated states by naringenin but not in the closed states [83]. Moreover, the ECG examinations suggested that the blockade of the hERG channel induced by naringenin results in prolongation of the QT interval [45]. Other authors showed that the addition of the antiarrhythmic drugs can strenghten the influence of naringenin on the hERG channel [84]. Nevertheless, such a combination may have an overstimulating effect and pose increased risk of arrhythmias.

**Table 2:** The effects of different flavonoids on the activity of Kv11.1 (hERG) channels. $EC_{50}$ is the concentration of a flavonoid that gives a half-maximal response. $IC_{50}$ is the concentration of a flavonoid concentration at 50% channel inhibition. The arrows symbolize the type of observed effects on the channel activity: ↓ inhibition, ↑ activation, → no effect. The table is in most part adapted from [81] with permission from Elsevier (2023).

Flavonoid	Type of Cell	Effect	$IC_{50}/EC_{50}$	References
Acacetin	HEK 293	↓	32.4μM	Li et al. [42] (2008)
Apigenin	Xenopus oocyte	→		Zitron et al. [82] (2005)
Chrysin	Xenopus oocyte	→		Zitron et al. [82] (2005)
Daidzein	HEK 293	↓		Zhang et al. [85] (2008)
7,8-Dimethoxyflavone	Xenopus oocyte	↓		Du et al. [86] (2015)
(−)-Epigallocatechin gallate	HEK 293	↓	6μM	Kelemen et al. [87] (2007)
	Xenopus oocyte	↓	20.5μM	
	CHO	↓		Kang et al. [44] (2010)
Fisetin	Xenopus oocyte	→		Zitron et al. [82] (2005)
	HEK 293	↓	38.4μM	Sun et al. [88] (2017)
Flavone	Xenopus oocyte	↓		Zitron et al. [82] (2005)
Galangin	Xenopus oocyte	→		Zitron et al. [82] (2005)
	HEK 293	↓		Sun et al. [88] (2017)
Genistein	HEK 293	↓		Zhang et al. [85] (2008)
Hesperetin	Xenopus oocyte	↓	289μM	Zitron et al. [82] (2005)
		↓	267μM	Scholz et al. [89] (2007)
Hesperidin	Xenopus oocyte	↑		Zitron et al. [82] (2005)
Isorhamnetin	HEK 293	↓		Sun et al. [88] (2017)
Kaempferol	Xenopus oocyte	↓		Zitron et al. [82] (2005)
	HEK 293	↓		Sun et al. [90] (2017)
Liquiritigenin	CHL	↓	53μM	Sweeney et al. [91] (2019)
Luteolin	HEK 293	↓		Sun et al. [88] (2017)
7,8-Methylenedioxyflavone	Xenopus oocyte	↓		Du et al. [86] (2015)
Morin	Xenopus oocyte	↓	111μM	Zitron et al. [82] (2005)
	HEK 293	↓		Sun et al. [88] (2017)
Myricetin	Xenopus oocyte	↑		Zitron et al. [82] (2005)
	HEK 293	→		Sun et al. [88] (2017)
Naringenin	Xenopus oocyte	↓	103μM	Scholz et al. [83] (2005)
		↓	173μM	Lin et al. [84] (2008)
		↓	102μM	Zitron et al. [82] (2005)
	HEK 293	↓	36.5μM	
	CHO	↓	35μM	Sanson et al.[45] (2022)
Naringin	Xenopus oocyte	→		Zitron et al. [82] (2005)
Neohesperidin	Xenopus oocyte	→		Zitron et al. [82] (2005)
Quercetin	Xenopus oocyte	↓		Zitron et al. [82] (2005)
	HEK 293	↓	12μM	Sun et al. [88] (2017)
Rutin	Xenopus oocyte	→		Zitron et al. [82] (2005)
	HEK 293	→		Sun et al. [88] (2017)
Taxifolin	HEK 293	→		Sun et al. [88] (2017)
Taxifolin 3-O-β-D-glucopyranoside	CHO	↓		Yun et al. [92] (2013)
Trimethylapigenin	HEK 293	↓	18–32μM	Liu et al. [38] (2012)

The other study showed the inhibitory effects of acacetin [42]. It was revealed that this flavonoid is not only able to block the Kv11.1 channel ($IC_{50}\approx$ 32 μM) but also suppress channel current through the recombinant human cardiac Kv7.1 with its regulatory subunit KCNE1, which plays a prominent role in the repolarization of cardiac action potential [93]. Surprisingly, this suppression does not induce QT prolongation syndrome.

Another study conducted by Kelemen and her collaborators [87] demonstrated that epigallocatechin-3-gallate, similarly to naringenin, is able to block the Kv11.1 channel both in the HEK 293 cells ($IC_{50}=6\;\mu M$) and in Xenopus oocytes ($IC_{50}=20.5\;\mu M)$. Nevertheless, in contrast to naringenin, the action of EGCG is reversible. Moreover, Kelemen’s research group showed that the inhibitory effects of epigallocatechin-3-gallate are slow and do not disappear completely after a wash-out, which suggests the long-term effect of this compound on channel gating. These results did not find confirmation in the study conducted by Kang et al. [44]. In contrast to Kelemen et al., they observed only mild inhibitory effects of this flavonoid acting on the CHO cells.

In the article [85], Zhang et al. showed that genistein can block the hERG channel in a reversible manner. The authors concluded that this inhibition is probably not direct and mediated by the protein tyrosine kinase mechanism. In that work, daidzein, which is a tyrosine kinase-inactive analog of genistein, turned out to be a substantially less potent inhibitor of the Kv11.1 channel.

Recently, Sun et al. [88] published their results concerning the impact of an ensemble of different flavonoids on the Kv11.1 channel expressed in HEK 293 cells. They found that the strongest inhibitory effects are exerted by quercetin ($IC_{50}=12\;\mu M$) and fisetin ($IC_{50}=38\;\mu M$). Luteolin turned out to be a little less potent with half-blocking concentration $IC_{50}>100\;\mu M$. The weak inhibitory effects were observed for other analyzed compounds, such as galangin, kaempferol, and isorhamnetin.

Du et al. [86] observed that the extract of *Galenia africana* L. (*Aizoaceae*) stem and leaves enables effective inactivation of the hERG channel. It turned out that this extract is more potent than its constituents 7,8-methylenedioxyflavone and 7,8-dimethoxyflavone applied alone. Thus, the authors concluded that this inhibitory effect may stem from some synergistic interaction action between several components of the extract.

In [38], Liu and collaborators studied the impact of the methylated derivative of apigenin, trimethylapigenin, on the Kv11.1 channels. As it turned out, in contrast to the apigenin itself [82], trimethylapigenin was able to suppress the activity of Kv11.1 channel in a fully reversible, concentration-dependent manner with $IC_{50}\approx 18\;\mu M$.

Another study carried out by Yun et al. [92] confirmed that also taxifolin 3-O-beta-D-glucopyranoside is able to effectively block the hERG channels in CHO cells. However, such inhibitory effects were not observed in terms of the administration of the taxifolin itself [90]. In addition, liquritigenin is able to inhibit the hERG channel at the moderate level ($IC_{50}\approx 53\;\mu M)$ by binding to the open state of the channel [91].

### 2.6. Further Kv Channels

Much less attention is paid in the literature to other types of Kv channels in the context of their modulation by the flavonoids. Let us provide the available information.

In [94], it was reported that the chronic administration of hesperetin was able to increase the expression of Kv1.2 channels in coronary arterial smooth muscle cells of diabetic rats. The authors conclude that since the expression of these ion channels is lowered in the diabetic rats, hesperetin can be considered a promising therapeutic agent in the treatment of coronary arterial dysfunction resulting from diabetes.

The flavonoids also influence the Kv7.1 channel, which contributes to the regulation of the repolarization phase of the cardiac action potential. Puerarin is an isoflavonoid found in the root of *Pueraria Lobata*, which is known from its anti-inflammatory, immunomodulatory, anti-cancer and cardioprotective properties [95]. In [43], it was demonstrated that the isoflavone, puerarin, effectively downregulates the channel activity via direct interaction with the channel protein. It was reported that this inhibitory effect (along with the blockade of the slow delayed rectifier current $I_{KS}$) contributed to the prolongation of action potential duration, which can be beneficial in the case of treatment of cardiovascular diseases. It turns out that also naringenin exerts an inhibitory effect on this channel, with a mild impact of this flavonoid on the $I_{KS}$ current [45]. Eventually, the studies performed by Kang et al. [44] revealed that also (−)-epigallocatechin-3-gallate [44] is a potent inhibitor of the Kv1.7 ion channel.

Quite recently, it has been discovered that another flavonoid, procyanidin B1, a natural compound extracted from the grape seed, is a potent inhibitor of the Kv10.1 channel ($IC_{50}=10\;\mu M$), which is overexpressed in some tumors [96]. According to this work, targeting the Kv10.1 channel by procyanidin B1 can inhibit the proliferation of cancerous cells. Consequently, this compound is a promising agent for cancer treatment.

## 3. Calcium-Activated Channels (KCa)

### 3.1. BK Channel

The large-conductance voltage- and $Ca^{2+}$-activated channels (BK) are ubiquitously expressed $K^{+}$ channels being characterized by a large single-channel conductance (150–300 pS) [97]. They are considered important drug targets due to their important roles in many physiological processes, such as neural transmission, hearing, endocrine secretion, and smooth muscle contraction [98]. In addition, the mitochondrial BK channel variants (mitoBK) received great scientific interest in terms of the possibilities of their chemical modulation because of the involvement of these channels in the regulation of metabolism, including ATP synthesis as well as the pro-life and pro-death processes [99,100,101].

**Figure 1:**
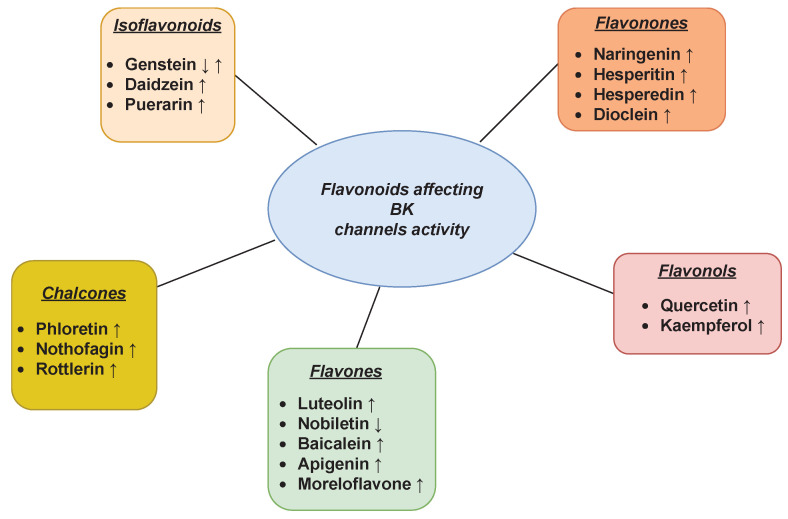
The schematic summary of the impact of key representatives of the main groups of flavonoids on the activity of the BK channels. Arrow up corresponds to the increase of the open state probability. Arrow down denotes channel inhibition. Both arrows represent the case when different types of channel modulation were reported depending on the cell types where the investigated BK channels were expressed.

The impact of flavonoids on the functioning of the BK channels was extensively studied in recent years. The main inferences from those investigations are outlined in Figure 1.

The activating effects were observed for the plasma membrane and mitochondrial BK channel variants in terms of the administration of a citrus flavanone, naringenin, in many different cell types [102,103,104,105,106,107,108,109,110]. The binding site for naringenin coordination seems to be located within the α subunits of the channel [103], probably within the gating ring. Naringenin coordination exerts the discernible effects on gating dynamics from the other stimuli [111] with no or negligible impact of auxiliary regulating β and γ subunits on naringenin binding. For this sake, it is anticipated that naringenin can be considered a general BK channel activator affecting all (or most) existing channel isoforms. According to [105], naringin exerts similar effects to naringenin on the BK channels. Another flavanone, dioclein, has been demonstrated to impose vasorelaxant effects, which can be, at least partially, explained by the activation of the BK channels and subsequent membrane hyperpolarization [112]. Among the flavanones, hesperedin also gained scientific interest as a modulator of $Ca^{2+}$-dependent channels. Namely, according to the in vitro study on the electrical activity of rat hippocampal cells [113], modulation of the BK channels is responsible for the anticonvulsive effects of hesperidin and its aglycone hesperetin.

Well-pronounced activating effects are reported in terms of the coordination of quercetin by the plasma-membrane BK channels exhibited in human bladder cancer cells, murine smooth muscles (ileal myocytes) and rat coronary smooth muscle cells [114,115,116]. In turn, the quercetin-related activation of the mitochondrial BK channel protein (mitoBK) was presented in human endothelial cell line EA.hy926 [117,118]. The physiological meaning of the quercetin-mediated BK and mitoBK channel activation is mainly associated with supporting the cell’s response to the oxidative stress [115] and cytoprotection [117]. Among other flavonols, the effective BK-opener profile was also shown for kaempferol in the work of Li et al. [119] in Xenopus oocytes injected with the mSlo gene. Moreover, the open-reinforcing impact of this flavonoid on the BK channel is responsible for relaxation of the rat pulmonary artery through the membrane hyperpolarization [120], which stays in agreement with the results obtained in [121]. In that work, the vasodilatory effects of kaempferol were related to its ability to stimulate the BK channels in human umbilical vein endothelial cells. The kaempferol-enhanced endothelium-dependent relaxation was observed in the porcine coronary artery, and it was also mediated by the activation of the BK channels [122].

Recent studies showed that luteolin acts as the mitoBK channel activator in cardiomyocytes and endothelial cells [123]. Luteolin-mediated mitoBK channel activation can contribute to the well-documented cardioprotective effects of this flavonoid. In contrast, another representative of flavones, nobiletin, can be considered a BK channel inhibitor, which acts in a voltage- and $Ca^{2+}$-dependent manner [124]. It is interesting that the presence of different regulating β subunits affects the efficacy of nobiletin action, which suggests that the inhibitory effects of this flavone can exhibit tissue selectivity, since the accessory BK channel subunits are frequently expressed in a tissue-dependent manner.

Considering the impact of other flavones on BK channels, in the studies on the relaxant effects of baicalein on tracheal smooth muscle [125], the authors anticipate that the molecular mechanism of bronchodilation induced by this substance incorporates the increase in the frequency of BK channels’ opening. Additionally, another flavone, apigenin, is an effective BK channel activator, according to the results obtained in [119] on Xenopus oocytes transfected with the mSlo gene. Among this group of flavonoids, the endothelium-dependent morelloflavone-induced vasorelaxation was observed in the experiments on isolated rat thoracic aorta, which was precontracted with norepinephrine [126]. This process partly involved the BK channels activation (and the opening of ATP-sensitive $K^{+}$ channels, as discussed in the next section).

Stimulation of the BK channels by isoflavonoid genistein resulted in both hampering and enhancing effects for the transport capabilities of the channel depending on the cell types under study. In the in vitro experiments on the selected molecular aspects of atherosclerosis described in [127], genistein inhibited the BK channels in the vascular smooth muscle cells stimulated by oxidized low-density lipoprotein. This kind of modulation resulted in the suppression of proliferation of those cells. The decrease of the BK channel activity in the presence of genistein (and genistein in combination with $Mg^{2+}$) was also observed in vascular smooth muscle cells in the rat model of hypertension [128] as well as in a similar rat model of hemorrhagic shock [129]. On the other hand, the voltage-dependent BK current was increased by this isoflavonoid in the case of the HEK 293 cells transfected by BK channels (i.e., human BK channel α and the β1 pcDNA3.1 plasmids) [130]. Moreover, the genistein-induced BK channel-activating effects were observed in bovine trabecular meshwork cells [131].

Another isoflavonoid, daidzein, acts as a concentration-dependent activator of the BK channel in complex with β1 subunit according to the results obtained in [132] in experiments carried out on rat cerebral basilar artery smooth muscle cells. Additionally, in the studies of vasorelaxation induced by genistein and daidzein in noradrenaline and KCl precontracted rat mesenteric artery preparations [133], it turned out that iberiotoxin (c= 1–10 nM) and charybdotoxin (c= 30 nM), being well-known antagonists of the BK channel, inhibited relaxation. These observations suggest activating effects of daidzein and genistein for BK channels in the case of the analyzed cells. The BK-stimulating effects of daidzein were confirmed in the investigations on Xenopus oocytes expressing mSlo [90]. However, better-pronounced effects were established in the case of channel stimulation by its analog, puerarin. Moreover, the authors observed the highest channel-activating potency of puerarin, when the BK channel was transfected in a form of mSlo–hβ1 complex, and this open-reinforcing effect can underlie the puerarin-mediated vasodilation [90]. Puerarin can also act as a mitochondrial BK channel modulator. According to the studies carried out on rat cardiomyocytes [134], pretreatment of the investigated cells with puerarin at c=0.24 mM for 5 min increased the cell viability against $H_{2}O_{2}$-stress. Further analysis indicated that the protection of cardiomyocytes against $H_{2}O_{2}$-stress by puerarin is mediated by the activation of mitochondrial BK channels. The mitoBK channel activation by puerarin was also observed in [135]. In that study, puerarin at c=0.24 mM protected rat myocardial cells from hypoxia/reoxygenation damage by enhancing the mitochondrial $K^{+}$ transport via mitoBK and mitoKATP channels (as discussed in the next section).

The open state probability of the BK channels in myelinated nerve fibers of Xenopus laevis was greatly increased by external phloretin (c= 10–200 μM). The analysis of the patch-clamp recordings of the BK channels stimulated by this chalcone showed that the open dwell times were prolonged and closed dwell times were shortened in relation to control data [136]. The action of phloretin, as a BK channel opener, was confirmed in [137,138], where the authors studied heterologously expressed BK channels composed of human α subunits in different concentrations of calcium ions and over a wide range of membrane potentials. Another chalcone, nothofagin, elicited endothelium-dependent vasodilation in the perfused rat kidney, and this effect is mediated by activation of the large-conductance potassium channels [139].

An alkaloid, berberine, is conditionally considered by some authors as an ‘isoquinoline flavonoid’ [140]. One of the cricial factors responsible for its biological meaning is BK channel modulation. According to the studies conducted on streptozotocin-induced diabetic rats [141], the chronic administration of berberine (100 mg/kg/day) can lower blood glucose level, reduce blood pressure and improve vasodilation. The important mechanism underlying that finding is that berberine markedly increased the open state probability and expression level of BK channels coordinated with β1-subunits in cerebral vascular smooth muscle cells isolated from diabetic rats or when exposed to hyperglycemia condition. Moreover, according to the research on *Sanoshashinto*, which is a classical prescription in China and Japan against hypertension, its key components, berberine and baicalin, are suggested to be responsible for the observed vasorelaxant effects [142]. It is hypothesized that the biological consequences of administration of these substances stem from the opening of the BK channels together with the activation of other pathways (the NO/cGMP and the DAG/PKC/CPI-17 pathway).

Another flavonoid that causes vasodilation is rottlerin. Such a physiological effect is mediated by rottlerin-induced BK channel activation, according to the rat and mouse models of cardioplegic arrest and reperfusion [143]. The open-reinforcing effect of rottlerin (in micromolar concentration) on BK channels was also observed in the studies performed on murine tracheal smooth muscles [144] (where it supported airway smooth muscle relaxation). The rottlerin-mediated BK channel activation was also detected in human hepatic stellate cells, where it was important for the liver profibrotic signaling pathways [145].

The summary of flavonoid modulation of BK channels activity is presented in Table 3.

### 3.2. IK and SK Channels

The small- and intermediate-conductance $Ca^{2+}$-dependent $K^{+}$ channels are not as extensively studied in terms of their effective stimulation by flavonoids as their large-conductance counterparts. Nevertheless, some reports emphasize the involvement of IK and SK channels in shaping the physiological response to flavonoid stimulation.

First, the molecular mechanism of vasodilation in rat aorta induced by quercetin is suggested to incorporate mainly activation of the SK channels, according to the works [146,147,148]. The SK channels play an important role in the cardioprotective effects of prolonged administration of an extract from leaves of Croton urucurana Baill. (*Euphorbiaceae*), which is popularly known as ‘sangue de dragão’, according to the spontaneously hypertensive rat model [149]. Flavonoids (including rutin, isoquercetin, kaempferol, vitexin) are the key bioactive substances in this extract [150]. The work [151] demonstrates that the vascular relaxation of rat aortic rings caused by a crude hydroalcoholic extract from Polygala paniculata (rich in rutin) involves the nitric oxide/guanylate cyclase pathway and subsequent opening of IK and BK channels. These effects are, however, larger in vitro than in vivo.

Among flavones, acacetin is an SK channel blocker, as confirmed in investigations on the small-conductance $Ca^{2+}$-dependent $K^{+}$ channels expressed in HEK 293 cells [152]. These studies evidenced that acacetin inhibited three subtypes of the SK channels (SK1, SK2, SK3) in a concentration-dependent manner with $IC_{50}$ of 12.4 μM for SK1, $IC_{50}$ = 10.8 μM for SK2, and $IC_{50}$ = 11.6 μM for SK3. The former experiments performed on a canine model (using isolated canine left atrium) showed that blockade of the SK channels by acacetin likely contributes to its anti-atrial fibrillation property [153]. Another flavone, isovitexin, obtained from the extract of Luehea divaricata Mart. regulates mesenteric arteriolar tone due to the activation of the SK channels and the Kir6.1 ATP-sensitive $K^{+}$ channels [154].

The studies on the vasorelaxant effects of genistein and daidzein administration [133] suggest that the activity of the SK channels can be modulated by these flavonoids and mediate the observed biological effects. The genistein- and daidzein-induced relaxation of rat noradrenaline precontracted arterial rings was decreased by apamin (c= 0.1–0.3 μM), being an antagonist of the SK channels.

Some studies on the effects of flavonoids on the activity of the $Ca^{2+}$-gated channels unraveled that although a given flavonoid modulates the activity of the BK channels, it does not interact with either SK or IK channels. Such an observation was made in the work of Xu et al. [120,121,122], where kaempferol had no effect on the SK and IK channels.

In turn, the studies of the vasodilatory properties of nothofagin [139] exclude the involvement of the SK channels in the mediation of that effect and indicate the main role of the BK channels. Nevertheless, a hypothesis on a potential additional contribution of the IK channels in the observed nothofagin-induced vasodilation cannot be rejected.

## 4. Inward Rectifying Potassium Channels (Kir)

The Inward Rectifying Potassium Channels (Kir) belong to one of the structurally simplest ion channels group containing four identical subunits, each containing two membrane-spanning alpha helices. These channels allow ions to be transported more effectively into than out of the cell. They are responsible for the regulation of resting membrane potential. Thus, their function is mostly related to the modulation of cardiac and neural cells activity, insulin secretion, or epithelial $K^{+}$ transport [155]. The Kir channels are expressed in many cell types: myocytes, neurons, blood cells, endothelial, glial cells, or oocytes [156]. The classification of the Kir channels family covers the groups Kir1–Kir7 together with their respective subgroups. Among the Kir channels, one can also distinguish the adenosine triphosphate (ATP)-dependent $K^{+}$ channels (KATP, Kir6) and the G-protein regulated $K^{+}$ channels (GIRK, Kir3). The structure of Kir channels lacks a proper voltage-sensing domain. Nevertheless, some representatives of the Kir family exert a bit stronger ‘’voltage dependence” than the others. In that aspect, Kir 2 channels, which are strongly rectifying ones (and consequently more sensitive to extracellular $K^{+}$), deserve to be distinguished.

The Kir channels can interact with a wide range of molecules, including flavonoids. Below, we shortly characterize the effects of flavonoid administration on the activity of inward rectifier potassium channels.

The literature indicates that flavones can affect the Kir channels’ activity. Jiao et al. [157] proved that flavones from rhododendron can stimulate the opening of ATP-dependent Kir channels in rat cardiomyocytes, which is related to the cardioprotective effects of this group of flavonoids. Another example of flavone being important in the context of Kir channel stimulation is luteolin. Li et al. [158] proved the positive impact of this compound for Kir channels present in rat coronary arterial smooth muscle cells, which was associated with inhibition of the process of vasoconstriction.

The flavonols represented by quercetin and rutin exert significant impact on Kir channels. Trezza et al. [159] examined the both modulators and 5-hydroxyflavone in the context of their possible impact on the ATP-sensitive Kir6.1 channel. They compared the experimental results of channels from *Rat norvegicus* aorta cells with molecular dynamics and docking calculations. All the compared results suggested that there was no effect on Kir6.1 caused by rutin, and significant downregulation in the case of quercetin and 5-hydroxyflavone, but only in the case of the closed channel conformation. The cardioprotective effect of flavonoids on rat myocytes through the regulation of mitochondrial ATP-sensitive potassium channels activity was also shown recently by Rameshrad et al. [160]. This research group studied a flavonol, morin, and postulated that its antioxidative effects are mediated by mitochondrial ATP-dependent potassium channels. The activity of Kir6.1 can be upregulated in the presence of isovitexin, which is obtained from the extract of Luehea divaricata Mart., according to [154]. This effect supports the regulation of mesenteric arteriolar tone.

In [161], the authors studied the physiological effects of the administration of Rooibos tea (Aspalathus linearis) as well as its pure flavonoid components: chrysoeriol, vitexin, and orientin. These substances were anticipated to mitigate hyperactive gastrointestinal disorders as well as exert health-beneficial effects in cardiovascular and respiratory diseases. With this aim, the research was conducted on fresh preparations of rabbit jejunum and aortic rings, guinea-pig trachea, and right atria. The main conclusion referred to the selective bronchodilator effect of Rooibos tea, which turned out to be mediated through KATP channel activation by chrysoeriol. This flavonoid also induced KATP-mediated relaxations of precontracted jejunum and aortic preparations by low (c= 25 mM) $K^{+}$ without any effect on high (c= 80 mM) $K^{+}$-induced contractions. Vitexin inhibited low $K^{+}$-induced contractions in jejunum and trachea, while orientin exerted only the relaxation effect in jejunum.

The in vitro studies on Xenopus oocytes expressing Kir6.2 channels showed that (−)-epigallocatechin-3-gallate and (−)-epicatechin-3-gallate (ECG) inhibit the activity of these ATP-sensitive potassium channels [162]. It turned out that ECG is three times more effective than EGCG. Two other compounds, (−)-epicatechin and (−)-epigallocatechin, did not affect the channel activity. Because the authors introduce structural modifications of the channel, in the conclusion, they formulate some hypotheses about the possible binding sited for EGCT within the Kir6.2 protein structure. In the same work, the authors analyze also the effects of EGCG on insulin secretory responses to high glucose loading in an in vivo rat model (hampering).

Naringenin has proven anti-inflammatory and antioxidant properties, which can be partly associated with the regulation of the ATP-sensitive potassium channels. In [163], Pinho et al. characterized this modulator in the context of activation of the NO–cyclic GMP–PKG–ATP-sensitive $K^{+}$ channel pathway, which can be related to the reduction of oxidative stress and translates into a decrease of inflammatory pain in mice. Similar results were obtained by Manchope et al. [164], where the activation of the same pathway leads to the reduction of the nociceptor hyperpolarization, and, in consequence, to the inhibition of its neuronal transmission. Another article that takes into consideration the function of ATP-sensitive channels in conjunction with the opioid receptors and the action of naringenin was written by [165], where the L-arginine/NO/cGMP/KATP pathway was analyzed. According to the studies performed on a rat model of ischemia–reperfusion (I–R) injury by Meng et al. [166], naringenin at a concentration above 2.5 μM activates KATP channels in both the plasma membrane and the mitochondria. In turn, the KATP channels’ activation contributes to the cardioprotective properties of naringenin.

Well-pronounced effects on the Kir channels (especially KATP channels) are also documented for a natural alkaloid, berberine (BBR), which is conditionally classified as an ’isoquinoline flavonoid’. BBR is frequently used in the Chinese and East Asian medicines [140]. Hua et al. confirmed the inhibition effect of BBR on ATP-sensitive channels [167]. The authors postulated that the anti-arrhythmic and antidiabetic properties of berberine are related to the inhibition of potassium channels. The inhibitory effects of berberine were also investigated by Wang et al. [168]. The authors characterized a similar anti-arrhythmic impact of BBR manifested by the reduction of action potential duration and the effective refractory period of ischemia. In contrast to BBR, a flavonoid from the anthocyanins-cyanidin caused the upreguation of Kir6.2 genes, which have potential implication in glucose sensitivity and its homeostasis [169]. Another flavonoid often used in Chinese and Japanese natural medicine is baicalein. The positive health aspects of baicalein is described in the context of potassium channels modulation by Saadat et al. [125]. In this work, the authors postulated that baicalein is an activator of ATP-dependent potassium channels in rat tracheal smooth muscle and is involved in bronchodilation through the promotion of the $K^{+}$ channel opening. Ribeiro et al. [170] suggest that the activation of the ATP-sensitive $K^{+}$ channels by baicalein can underlie the gastroprotective properties of this flavonoid.

A group of flavonoids with a strong influence on the Kir channels is isoflavonoids. Among them, genistein is considered the most common modulator of inwardly rectifying potassium channels. This compound exhibited a typical Kir channels inhibiting profile in several studies. Zhao et al. [171] described the molecular character of this inhibition. For the Kir2.3 channel, it was proved that the key protein regions responsible for the genistein-related inhibition are transmembrane domains and the pore. Ko et al. [172] showed that genistein blockade depends on the mode of the channel activity—the modulator did not exert any effect on the steady-state activation or inactivation of Kir channels. In the work written by Okamoto et al. [173], it was shown that the reduction of the Kir current induced by genistein entailed the depolarization of membrane of rat osteoclast, and in final effect, it caused an elevation of $Ca^{2+}$ and inhibition of osteoclastic bone resorption. The earlier work by Okata et al. [174] had suggested that the tyrosine kinase may be involved in the inhibitory character of the impact of genistein on ATP-dependent channels.

Another isoflavonoid, puerarin, exerts an activating effect on the mitochondrial $K^{+}$ ATP-regulated channels (mitoKATP), according to the results presented in [135]. That study concluded that the mitoKATP channel activation participated in the cardioprotection by puerarin. The activation of mitochondrial KATP channels also plays a crucial role in shaping the cardioprotective effects exerted by other flavonoids [175]. Among them, six natural compounds should be mentioned: (−)-epigallocatechin-3-gallate [176], theaflavin [177], proanthocyanidins [178], genistein [179], baicalein [180], and morin[160].

As one can see, flavonoids can be considered KATP channel modulators. The summary of flavonoid modulation of ATP-sensitive Kir channels is outlined in Table 4.

Yow et al. [182] characterize the impact of naringin as a direct activator of the G protein-coupled Kir channel, which is important in CNS control and heart rate regulation. A flavanone, hesperidin, interacts with the G protein-activated GIRK1 and GIRK2 channels and causes their inhibition, according to the results presented in [183]. It turns out that hesperidin inhibits GIRK1 and GIRK2 currents through binding to the μ-opioid receptor, and it participates in the anti-depressant and antinociceptive activities of hesperidin. The GIRK current may be also inhibited by eriodictyol, from a flavanone group, which occurs in citrus fruits and Chinese herbs. The inhibition character of this flavonoid on GIRK channels was documented in the work of Hammadi et al. [184]. The impact of flavonoids on the G-protein activated Kirs is summarized in Table 5.

## 5. Two-Pore Domain Potassium Channels (K2P)

The two-pore domain potassium channels are widely distributed in excitable and non-excitable cells and are responsible for the background potassium conductance [185,186]. They are emerging drug targets in case of a.o. cardiovascular and neurological diseases [187,188,189,190]. The K2P channels are a family of 15 $K^{+}$ channel subtypes, including the TWIK channels (named as an acronym for Tandem of pore domains in a Weak Inward rectifying $K^{+}$ channels) $K_{2p}1.1$, $K_{2p}6.1$ and $K_{2p}7.1$, TREK channels (TWIK-related $K^{+}$ channels): $K_{2p}2.1$ and $K_{2p}10.1$, TRAAK channel (TWIK-related arachidonic acid-activated potassium channel) belonging to the TREK subgroup: $K_{2p}4.1$, TASK channels (TWIK-related acid-sensitive $K^{+}$ channel channels): $K_{2p}3.1$, $K_{2p}5.1$, $K_{2p}9.1$ and $K_{2p}15.1$, THIK channels (tandem pore domain halothane-inhibited $K^{+}$ channels): $K_{2p}12.1$ and $K_{2p}13.1$, TALK channels (TWIK-related alkaline pH-activated $K^{+}$ channels): $K_{2p}16.1$ and $K_{2p}16.1$, and TRIK channel (TWIK-related spinal cord $K^{+}$ channel) $K_{2p}18.1$.

Among the TREK subgroup of K2P channels, TREK-1 ($K_{2p}2.1$) and TRAAK ($K_{2p}4.1$) are mainly expressed in the central nervous system (CNS), and TREK-2 ($K_{2p}10.1$) is expressed in both CNS and peripheral tissues [191,192]. TREK channels are activated by several stimuli, including biomolecules (e.g., riluzole, nitrous oxide, polyunsaturated fatty acids, and lysophospholipids). These modulators can contribute to the opening of TREKs under pathological conditions. Considering the effects of flavonoids’ administration, the neuroprotective properties of quercetin were demonstrated in [193]. In that study, the mice manic model was induced by i.p. injection of D-amphetamine, and quercetin suppressed the neural excitability of prefrontal cortex pyramidal neurons. This effect was mediated by enhancing current flow through TREK-1 channels, which decreased membrane resistance. In [194], the authors demonstrate that baicalein and wogonin increased the open state probability of TREK-2 channels in a dose-dependent manner (in the range from 0 to 100 μM, at which the maximal channel-activating effect was observed), leaving the single-channel conductance and mean open dwell-time unchanged. These studies were carried out on the COS-7 cells (African green monkey kidney fibroblast-like cell line) transfected with rat TREK-2. Since baicalein elicited a continuous channel-activating effect, while wogonin activated the TREK-2 channel transiently, it was anticipated that these flavones interact with the TREK-2 channel protein by different molecular mechanisms. Nevertheless, the TREK-2 modulation by wogonin and baicalein may exert beneficial effects in neuroprotection.

The studies on the possible impact of tyrosine kinase inhibitor, genistein, on the activity of the human TASK-1 ($K_{2p}3.1$) channel expressed in Xenopus oocytes and Chinese hamster ovary cells (CHO) revealed the blocking effects ($IC_{50}$ = 10.7 μM in Xenopus oocytes and $IC_{50}$ = 12.3 μM in CHO cells) [195,196]. These studies showed that an isoflavonoid, daidzein (at a concentration of 100 μM), causes 18.2 ± 1.3% inhibition of human TASK-1 expressed in Xenopus oocytes [196]. Moreover, the TASK-3 ($K_{2p}9.1$), THIK-1 ($K_{2p}13.1$) and TWIK-2 ($K_{2p}6.1$) currents also decreased in the presence of genistein [195,196], and the same relation for the TASK-2 ($K_{2p}5.1$) activity was suggested in [197]. These observations allowed one to make the inference that inhibition of the K2P currents via biochemically induced changes in tyrosine kinase activity permits membrane potential depolarization and excitation.

## 6. Discussion

**Figure 2:**
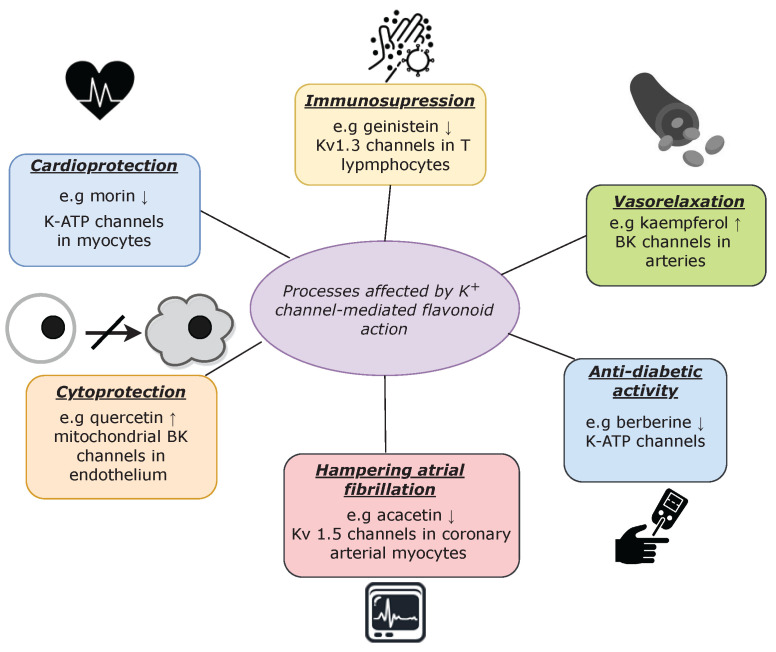
A graphical representation of the selected important biological processes that can be affected by flavonoid administration, for which the molecular control mechanism can be, at least, partially explained by the $K^{+}$ channel modulation (with appropriate examples). The ↑ represents channels’ activation and ↓ denotes channels’ inhibition.

Flavonoids are widely known for their beneficial health effects, which involve complex biochemical interactions with specific molecular targets, including potassium channels. Due to the fact that these transport proteins play important roles in shaping cardiac action potential as well as smooth muscle tone, their stimulation by flavonoids yields vasorelaxant and cardioprotective effects [81,148,175,198,199,200]. Nevertheless, these effects are not the only examples of the $K^{+}$ channel-mediated physiological processes that are regulated by flavonoids, as presented in Figure 2.

In this work, we summarized the state of knowledge about flavonoids as modulators of particular subtypes of $K^{+}$ channels. It allowed us to point out the most promising natural substances for further research from the pharmacological point of view. Such future studies can include the analysis of their derivatives to develop novel substances which may exert better specificity and efficiency against particular channel proteins. However, this is a challenging task due to the complex mechanisms of flavonoid interactions with channel proteins (direct and/or indirect via second-messenger proteins or the changes of membrane properties) as well as multiple possibilities of the structure–function modification. As an example, let us refer to the studies on quercetin being an effective mitoBK channel activator and its analog isorhamnetin which turned out to not affect the mitochondrial BK channel activity [118]. On the other hand, the relatively weak inhibitory effect of the apigenin on the Kv1.5 channel is improved when the methylated derivatives of this flavonoid (dimethylapigenin or trimethylapigenin) are introduced at the same concentrations [68]. Furthermore, the enhanced inhibition of the Kv 1.3 channel was observed when the active compounds possess a prenyl group in its structure in comparison to their non-prenylated counterparts (such as 8-prenylnaringenin, isoxanthohumol in relation to their non-prenylated analogs naringenin, genistein) [31,32].

As can be observed in Table 1, Table 2, Table 3, Table 4 and Table 5, some flavonoids (e.g., naringenin, quercetin, genistein) have a wide spectrum of molecular targets within the family of $K^{+}$ channels. It suggests the existence of multiple possible binding sites for these biomolecules within channel protein structures or a number of second-messenger molecules in case of indirect mechanisms or the relatively large effects exerted by the physicochemical modulation of membrane properties by flavonoids, which accounts for the flavonoid–channel interactions. According to the literature, the last factor, i.e., the interactions of flavonoids with the membrane, and the consequent changes of the membrane composition, packaging, fluidity, permeability and interactions of its lipid components with the membrane proteins [201,202,203] significantly modify membrane-mediated cell signaling cascades. Thus, it is partly responsible for the pharmacological activities of flavonoids, including its anti-tumor, anti-microbial and anti-oxidant properties [204,205,206,207]. The effects exerted by flavonoids on the biological membranes are mainly associated with their planar structure and lipophilicity, which are dependent on, among others, the number and position of hydroxyl groups [201,203]. Relatively hydrophobic flavonoids (such as flavones) are incorporated into the interior of the lipid bilayers, and they increase the ordering and dynamics within the internal (fatty) part of the membrane. In contrast, more hydrophilic flavonoids (such as flavonols) interact primarily with the membrane surface. The localization and strength of the flavonoids–membrane interactions affect the functioning of integral membrane proteins (including ion channels) and modulate their structure and function [208,209,210,211]. That is because the membrane proteins strongly interact with their lipid surroundings. They are not rigid entities, but to ensure a good hydrophobic matching to the adjacent lipid bilayer, they undergo structural deformations. Consequently, due to such deformations, any change of the membrane properties can result in the allosteric modulation of ion channels functioning. From this perspective, considering the molecular mechanisms of flavonoid–channel interactions, there exists a strong correlation between the structure and the molecular activity of a flavonoid. It stems from the synergistic effects of the effective and specific binding of a given flavonoid to a particular target protein (e.g., ion channel) and the additional indirect interactions mediated by the membrane. As an example, the differences in lipid composition in cell membrane and mitochondrial membranes are anticipated to contribute to the possible quantitative differences in the flavonoid-mediated activation levels of plasma-membrane and mitochondrial variants of potassium channels (e.g., BK/mitoBK channels) [203]. Analogous effects can make a contribution to the quantitative differences between the outcomes of channel activation by a given flavonoid in different cell types.

Due to the existing structural and mechanistic differences implying other binding sites for flavonoids or various paths of indirect interactions, even if a given flavonoid can regulate different channel types, the directions of these modulations can be completely different. For instance, quercetin is an activator of the BK channels (from plasma membrane and their mitochondrial analogues) [114,115,116,117,118], but it has an inhibitory effect on the Kir6.1 channels [159]. Analogously, naringenin stabilizes the open state of the BK and mitoBK channels [102,103,104,105,106,107,108,109,110]. At the same time, it has a multichannel inhibitory profile against hERG, Kir2.1, Kv7.1, and Kv4.3 channels [45].

For some flavonoids, many details of the molecular mechanism of their specific interactions with channel proteins become unraveled, as in the case of naringenin and quercetin coordination to the plasma membrane/mitochondrial BK channels [103,111,118] or Kir6.1 modulation caused by quercetin and 5-hydroxyflavone [159]. Nevertheless, a clear picture of the possible direct or indirect interactions between most flavonoids and channel types remains unknown. Therefore, it can become a field of exploitation for both experimental and in silico studies.

Apart from the reports mentioned before within this review, which precisely describe the effects of flavonoid administration on particular subtypes of potassium channels, in the literature, one can also encounter the ones that provide only general information about the involvement of $K^{+}$ channels in mediating the flavonoid-induced physiological effect. In such studies, either a non-specific $K^{+}$ channel blocker (tetraethylammoniumchloride) or combinations of different blockers were applied in the experimental work. Let us provide a few examples. Sinensetin from Orthosiphon stamineus Benth. (*Lambiaceae*) results in vasorelaxation. In turn, the strong inhibition of the vasorelaxant effects elicited by this flavonoid was observed in terms of administration of the potassium channel antagonists, which suggests the employment of different pathways involving the Kir, KCa and Kv channels in the considered phenomenon [212]. Analogous vasorelaxant effects mediated by $K^{+}$ channels were observed for the rat aortic rings treated with luteolin [213]. The investigations on biochemical responses in terms of the cellular stress induced by apigenin isolated from Aster yomena in Candida albicans [214] indicated that apigenin induced ion channel-mediated potassium leakage. In turn, the important role of potassium channels in the attenuation of neurotoxic mitochondrial calcium overload by a Citrus polymethoxylated flavone, nobiletin, was suggested in [215]. Another study, which focuses on the antidepressant-like effect of hesperidin in a Tail Suspension Test in mice, demonstrated the contribution of the KATP and KCa channels in this phenomenon [216]. Moreover, hesperidin and its aglycone, hesperetin, are associated with beneficial outcomes for human health, such as prevention of cancer and counteracting cardiovascular diseases, which is partly due to the $K^{+}$ channel modulation according to [217]. Thus, to extract the exact information about the particular types of potassium channels affected by the mentioned flavonoids, further research is needed.

Considering the other interesting directions for further research, we are convinced that a thorough extended analysis of the physiological pathways (possibly, partly mediated by the $K^{+}$ channels) responsible for the antidiabetic, anti-inflammatory, and anti-cancerogenic effects of the flavonoids’ administration could be recommended. An additional interesting approach is to synthesize hybrid molecules made of two different substances with synergistic action in modulating potassium channels. As an example, this approach has been successfully realized in the synthesis of celecoxib with the flavonoid combrestatin A-4 with the goal to improve the anti-inflammatory properties of both substances[218]. This could yield valuable contributions as a response to the popular and challenging public health problems worldwide.

There are already some reports on the regulation of insulin homeostasis and metabolic processes by flavonoids, as summarized in [219]. In this work, we have also mentioned the studies which associate the antidiabetic effects of berberine with KATP channels’ inhibition [167]. Moreover, according to [141], this substance counteracts the diabetes-related vascular complications via BK channel activation in cerebral smooth muscle cells. In the antidiabetic context, naringenin (repeatedly mentioned in this review as a $K^{+}$ channel modulator) also deserves particular attention [220,221]. The metabolic metabolic diseases including obesity, metabolic syndrome and type 2 diabetes (T2D) are gathered by the excess adiposity, which sustains a state of chronic low-grade inflammation. In turn, chronic inflammation is an important factor contributing to DNA damage and can lead to cancer [222,223]. From this perspective, the administration of flavonoids, which exhibit a relatively wide spectrum of beneficial effects, such as promoting anti-oxidation (as mentioned in case of the, e.g., mitochondrial BK channel modulation) and immunosuppression (as discussed in Section 2), seems reasonable especially for the high-risk populations, e.g., suffering T2D [220,221] or endocrinopathies [224,225,226].

Another interesting aspect of flavonoid delivery is the alleviation of the effects of hormonal imbalance [224,225,226]. For instance, genistein and daidzein are estrogen-like compounds, xenoestrogens, that can bind competitively to estrogen receptors. Thus, they are considered an alternative to hormone replacement therapy in postmenopausal women or patients with some ovary dysfunctions [227]. What is worth mentioning is that some of their beneficial effects are mediated by $K^{+}$ channels. In general, the usage of phytoestrogens can be also recommended from the perspective of the possible neuroprotection [228], prevention of cancer [229], and atherosclerosis [133]. Still, the determination of the direct effects of flavonoid xenoestrogens on the endocrine system can be an interesting subject for further research.

To sum up, the molecular aspects of the prophylactic and therapeutic effects of flavonoids can become a promising field of exploitation for further biochemical and pharmacological investigations. This direction of research could provide a scientific justification for the flavonoid supplementation with the aim of prevention and treatment of popular diseases, where prolonged conventional therapy can be burdensome for patients and exhibit side effects.

## 7. Conclusions

Flavonoids are a group of natural substances that can effectively interact and regulate the functioning of many potassium channel types, which has been outlined in this review. The modulation of $K^{+}$ channels by flavonoids and their derivatives, together with their physiological consequences, should be subject to further investigation as a promising approach to the prevention or treatment of, among others, cardiovascular and inflammatory diseases.

## Figures and Tables

**Table 1:** Effects of flavonoids on the activity of different subtypes of the Kv channels. HTL stands for human T lymphocytes. HLTJ is human leukemic T cells. $EC_{50}$ is defined as the concentration of a flavonoid that gives the half-maximal response. $IC_{50}$ is the concentration of a flavonoid concentration at 50% channel inhibition. The arrows symbolize the type of observed effects on the channel activity: ↓ inhibition, ↑ activation, → no effect. As an exception, the table includes resveratrol, which is not a flavonoid, but it was analyzed in the same series of experiments as flavonoids.

Kv Channel Subtype	Flavonoid	Type of Cell	Effect	${\mathrm{IC}}_{50}/{\mathrm{EC}}_{50}$	References
Kv1.3	Genistein	HTL	↓	30–60μM	Teisseyre et al. [25] (2005)
	Daidzein	HTL	→		Teisseyre et al. [25] (2005)
	6-Prenylnaringenin	HLJT	↓	5.8μM	Teisseyre et al. [26] (2018)
	Acacetin	HLJT	↓	30μM	Teisseyre et al. [26] (2018)
			↓	21μM	Zhao et al. [27] (2014)
	Chrysin	HLJT	↓	26 μM	Teisseyre et al. [26] (2018)
	Chrysin + mevastatin	HLTJ	↓	8 μM	Teisseyere et al. [28] (2022)
	Chrysin + simvastatin	HLTJ	↓	11μM	Teisseyere et al. [28] (2022)
	Baicalein	HLJT	→		Teisseyre et al. [26] (2018)
	Wogonin	HLJT	→		Teisseyre et al. [26] (2018)
	Luteolin	HLJT	→		Teisseyre et al. [26] (2018)
	Resveratrol	HTL	↓	41μM	Teisseyre et al. [29] (2006)
	Naringenin	HTL	→		Teisseyre et al. [30] (2009)
	Naringenin-4${}^{\prime }$,7-dimethylether	HTL	↓		Teisseyre et al. [30] (2009)
		HLTJ	↓	13μM	Gąsiorowska et al. [31] (2015)
	Naringenin-7-methylether	HTL	↓		Teisseyre et al. [30] (2009)
		HLJT	↓	16μM	Gąsiorowska et al. [31] (2009)
	Aromadendrin	HTL	→		Teisseyre et al. [30] (2009)
	Isoxanthohumol	HLJT	↓	7.8μM	Gąsiorowska et al. [31] (2015)
	Xanthohumol	HLJT	↓	3.1μM	Gąsiorowska et al. [31] (2015)
	8-prenylnaringenin	HLJT	↓		Gasiorowska et al. [32] (2012)
	Licochalcone A	HLJT	↓	0.83μM	Phan et al. [33] (2021)
	8-prenylnaringenin+mevastatin	HLTJ	↓	7μM	Teisseyre et al. [28] (2022)
Kv1.5	Myricetin	HEK 293	↓		Ou et al. [34] (2016)
	Hesperetin	HEK 293	↓	23μM	Wang et al. [35] (2016)
	Quercetin	Xenopus oocytes	↑	37.8μM	Yang et al. [36] (2009)
		rats (*in vivo*)	↑		Morales-Cano et al. [37] (2014)
		HEK 293	↓		Liu et al. [38] (2012)
	3,7,3${}^{\prime }$,4${}^{\prime }$-tetramethylquecertin	HEK 293	↓		Liu et al. [38] (2012)
	3,5,7,3${}^{\prime }$,4${}^{\prime }$-pentamethylquecertin	HEK 293	↓		Liu et al. [38] (2012)
	Apigenin	HEK 293	↓		Liu et al. [38] (2012)
	7,4${}^{\prime }$-dimethylapigenin	HEK 293	↓		Liu et al. [38] (2012)
	5,7,4${}^{\prime }$-trimethylapigenin	HEK 293	↓	6.4μM	Liu et al. [38] (2012)
	EGCG	CHO	↓	101μM	Choi et al. [39] (2001)
	Isoliquiritigenin	H9c2	↓		Noguchi et al. [40] (2008)
	Acacetin	HEK 293	↓		Wu et.al [41] (2011)
		atrial myocytes	↓	3.2μM	Li et al. [42] (2008)
Kv1.7	Puerarin	HEK 293	↓	36μM	Xu et al. [43] (2016)
	(−)-Epigallocatechin-3-gallate	CHO	↓	30μM	Kang et al. [44]
	Naringenin	CHO	↓	110μM	Sanson et al. [45] (2022)
Kv2.1	Isoliquiritigenin	H9c2	↓	0.11μM	Noguchi et al. [40] (2008)
	Genistein	HEK 293	↓		Aréchiga-Figueroa et al. [46] (2017)
	Naringenin-4${}^{\prime }$,7-dimethylether	CHO	↓	21μM	Gu et.al [47] (2022)
Kv4.3	Genistein	CHO	↓	125μM	Kim et al. [48] (2011)
	Daidzein	CHO	↓		Kim et al. [48] (2011)
	Genistin	CHO	→		Kim et al. [48] (2011)
	Epigallocatechin-3-gallate	CHO	↓		Kang et al. [44] (2010)
	Naringenin	CHO	↓	115μM	Sanson et al. [45] (2022)
	5,7,4${}^{\prime }$-trimethylapigenin	human atrial myocytes	↓	19.8μM	Liu et al. [38] (2012)
Kv10.1	Procyanidin B1	HEK	↓	10μM	Na et al. [49] (2020)

**Table 3:** The effects of different flavonoids on the activity of BK channels’ isoforms in different cell types. The ↑ represents channel activation, and ↓ denotes channel inhibition. The table includes the effects of berberine, which does not strictly belong to the flavonoid family. Nevertheless, by some authors, it is categorized as ’isoquinoline flavonoid’.

Flavonoid	Material	Effect	References
Naringenin	rat aortic rings	↑	Saponara et al. [102] (2006)
	HEK 293T	↑	Hsu et al. [103] (2014)
	colonic smooth muscle cells	↑	Yang et al. [104] (2014)
	rat tracheal smooth muscle cells	↑	Shi et al. [105] (2019)
	mitoplasts from rat heart (left ventricular tissue)	↑	Tesai et al. [106,107] (2013, 2017)
	mitoplasts from primary human dermal fibroblasts	↑	Kampa et al. [108] (2019)
	mitoplasts from human endothelial cells EA.hy926	↑	Kicinska et al. [109] (2020)
Naringin	rat tracheal smooth muscle cells	↑	Shi et al. [105] (2019)
Dioclein	rat small mesenteric arteries	↑	Cortes et al. [112] (2001)
Hesperidin	rat hippocampal cells	↑	Dimpfel et al. [113] (2006)
Hesperetin	rat hippocampal cells	↑	Dimpfel et al. [113] (2006)
Quercetin	human bladder cancer cells	↑	Kim et al. [114] (2011)
	murine smooth muscles (ileal myocytes)	↑	Melnyk et al. [115] (2019)
	rat coronary smooth muscle cells	↑	Zhang et al. [116] (2020)
	mitoplasts from human endothelial cells EA.hy926	↑	Kampa et al. [117,118] (2021, 2022)
Kaempferol	Xenopus oocytes	↑	Li et al. [119] (1997)
	human umbilical vein endothelial cells	↑	Xu et al. [121] (2008)
	porcine coronary artery	↑	Xu et al. [122] (2015)
	rat pulmonary artery	↑	Mahobiya et al. [120] (2018)
Luteolin	mitoplasts from rat cardiomyocytes, mitoplasts from human endothelial cells EA.hy926	↑	Kampa et al. [123] (2022)
Baicalein	rat tracheal smooth muscle	↑	Saadat et al. [125] (2019)
Apigenin	Xenopus oocytes	↑	Li et al. [119] (1997)
Morelloflavone	rat thoracic aorta	↑	Lamai et al. [126] (2013)
Genistein	rat vascular smooth muscle cells	↓	Bai et al. [127] (2020)
	vascular smooth muscle cells	↓	Sun et al. [128] (2015)
	rat superior mesenteric artery	↓	Zhou et al. [129] (2005)
	HEK 293 cells	↑	Wang et al. [130] (2017)
	rat mesenteric artery rings	↑	Nevala et al. [133] (2001)
	bovine trabecular meshwork cells	↑	Stumpff et al. [131] (1999)
Daidzein	rat cerebral basilar artery smooth muscle cells	↑	Zhang et al. [132] (2010)
	Xenopus oocytes	↑	Sun et al. [90] (2007)
	rat mesenteric artery rings	↑	Nevala et al. [133] (2001)
Puerarin	Xenopus oocytes	↑	Sun et al. [90] (2007)
	mitochondria of rat cardiomyocytes	↑	Yang et al. [134] (2008)
	mitochondria of rat cardiomyocytes	↑	Yao et al. [135] (2010)
Phloretin	myelinated nerve fibres of Xenopus laevis	↑	Koh et al. [136] (1994)
	heterologous expression models (unspecified in the cited work)	↑	Gonzalez et al. [137,138] (2012, 2013)
Nothofagin	rat kidney cells	↑	Marques et al. [139] (2020)
Berberine	cerebral vascular smooth muscle cells	↑	Ma et al. [141] (2017)
Rottlerin	murine tracheal smooth muscle	↑	Goldklang et al. [144] (2013)
	human hepatic stellate cells	↑	Yang et al. [145] (2020)

**Table 4:** The effects of flavonoids on the KATP channels. The ↑ represents channel activation and ↓ denotes channel inhibition, while → stands for no effect on channel activity.

KATP Channels	Flavonoid	Cell Type	Effect	References
Kir6.1	Quercetin	Rat norvegicus aorta/MD	↓	Trezza et al. [159] (2018)
	5–Hydroxyflavone	Rat norvegicus aorta/MD	↓	Trezza et al. [159] (2018)
	isovitexin	rat isolated mesenteric beds	↑	Tirloni et al. [154] (2019)
Kir6.2	Cyanidin	Rat Pancreatic β-cells INS-1	↑	Suantawee et al. [169] (2017)
	(−)-Epigallocatechin-3-gallate	Xenopus oocytes	↓	Jin et al. [162] (2007)
	(−)-Epicatechin-3-gallate	Xenopus oocytes	↓	Jin et al. [162] (2007)
	(−)-Epicatechine	Xenopus oocytes	→	Jin et al. [162] (2007)
	(−)-Epigallocatechin	Xenopus oocytes	→	Jin et al. [162] (2007)
Kir6.x	Berberine	Guinea pig ventricular myocytes	↓	Hua Z et al. [167] (1994)
Kir6.x	Berberine	Guinea pig ventricular myocytes	↓	Wang et al. [168] (1996)
Kir6.x	Naringenin	myocardial cells of Sprague-Dawley rats	↑	Meng et al. [166] (2016)
Kir6.x	Naringenin	Human Umbilical Vein	↑	Protic et al. [181] (2014)
Kir6.x	Baicelin	Rat tracheal smooth muscle	↑	Saadat et al. [125] (2019)
Kir6.x	TFR	Gat cardiomyocytes	↑	Jiao Li et.al. [157] (2015)
Kir6.x	Genistein	Rabbit portal vein smooth muscle	↓	Ogata et.al. [174] (1997)
Kir6.x	Baicalein	Mice gastric mucosal ulcerations	↑	Ribeiro et.al. [170] (2016)
Kir6.x	Morin	Mitoplasts from rat myocardial cells	↑	Rameshrad et.al. [160] (2021)
Kir6.x	Chrysoeriol	rabbit jejunum and aortic rings, guinea-pig trachea	↑	Khan et.al. [161] (2006)
Kir6.x	Vitexin	rabbit jejunum, guinea-pig trachea	↑	Khan et.al. [161] (2006)
Kir6.x	Orientin	rabbit jejunum	↑	Khan et.al. [161] (2006)
mitoKATP	Puerarin	Rat cardiomyocytes	↑	Yao et al. [135] (2012)
mitoKATP	Naringenin	Rat cardiomyocytes	↑	Meng et al. [166] (2016)
mitoKATP	Baicalein	Chicken embryonic cardiomyocyte	↑	Tu et al. [180] (2008)
mitoKATP	(−)-Epigallocatechin-3-gallate	Rat cardiomyocytes	↑	Song et al. [176] (2010)
mitoKATP	Theaflavin	Rat cardiomyocytes	↑	Ma et al. [177] (2011)
mitoKATP	Proanthocyanidins	Rat cardiomyocytes	↑	Hu et al. [178] (2014)
mitoKATP	Genistein	Rabbit cardiomyocytes	↑	Yao et al. [179] (2009)
mitoKATP	Morin	Rat cardiomyocytes	↑	Yao et al. [160] (2021)

**Table 5:** The effects of flavonoids on the GIRK channels. The ↑ represents channel activation and ↓ denotes channel inhibition.

GIRK Channels	Flavonoid	Cell Type	Effect	References
Kir3.1/Kir3.2	Hesperidin	Xenopus laevis oocytes	↓	Loscalzo et al. [183] (2011)
Kir3.1/Kir3.4	Eriodictyol	HEK-293 (human embryonic kidney)	↓	Hammadi et al. [184] (2019)
Kir3	Naringin	Xenopus laevis oocytes	↑	Yow et al. [182] (2011)

## Data Availability

Not applicable.

## Abbreviations

The following abbreviations are used in this manuscript:

AF	atrial fibrillation
AP	action potential
BK	big-conductance (large-conductance) $Ca^{2+}$-dependent potassium channels
CNS	central nervous system
$EC_{50}$	half maximal effective concentration
EGCG	(−)-epigallocatechin-3-gallate
$IC_{50}$	inhibitory concentration 50%
IK	intermediate-conductance $Ca^{2+}$-dependent potassium channels
$I_{Kur}$	ultra-rapid delayed rectifier current
K2P	two-pore domain potassium channels
KCa	$Ca^{2+}$-regulated potassium channels
Kv	voltage-regulated (voltage-gated) potassium channels
Kir	inward rectifier potassium channels
mitoKATP	mitochondrial $K^{+}$ ATP-regulated channels
mitoBK	mitochondrial BK channels
PH	pulmonary hypertension
SK	small-conductance $Ca^{2+}$-dependent potassium channels
T2D	type 2 diabetes
TMDs	transmembrane domains
TALK	TWIK-related alkaline pH-activated $K^{+}$ channels
TASK	TWIK-related acid-sensitive $K^{+}$ channel channels
THIK	tandem pore domain halothane-inhibited $K^{+}$ channels
TRAAK	TWIK-related arachidonic acid-activated potassium channel
TREK	TWIK-related $K^{+}$ channels
TRIK	TWIK-related spinal cord $K^{+}$ channel
TWIK	tandem of pore domains in a weak inward rectifying $K^{+}$ channels
